# Recent advances in understanding the molecular basis of melanogenesis in melanocytes

**DOI:** 10.12688/f1000research.24625.1

**Published:** 2020-06-15

**Authors:** Norihiko Ohbayashi, Mitsunori Fukuda

**Affiliations:** 1 Faculty of Medicine and Graduate School of Comprehensive Human Sciences, University of Tsukuba, Tsukuba 305-8575, Japan; 2 Laboratory of Membrane Trafficking Mechanisms, Department of Integrative Life Sciences, Graduate School of Life Sciences, Tohoku University, Miyagi 980-8578, Japan

**Keywords:** BLOC, melanogenic enzymes, melanosome maturation, membrane traffic, Rab small GTPase

## Abstract

Melanin pigments are responsible for human skin and hair color, and they protect the body from harmful ultraviolet light. The black and brown melanin pigments are synthesized in specialized lysosome-related organelles called melanosomes in melanocytes. Mature melanosomes are transported within melanocytes and transferred to adjacent keratinocytes, which constitute the principal part of human skin. The melanosomes are then deposited inside the keratinocytes and darken the skin (a process called tanning). Owing to their dark color, melanosomes can be seen easily with an ordinary light microscope, and melanosome research dates back approximately 150 years; since then, biochemical studies aimed at isolating and purifying melanosomes have been conducted. Moreover, in the last two decades, hundreds of molecules involved in regulating melanosomal functions have been identified by analyses of the genes of coat-color mutant animals and patients with genetic diseases characterized by pigment abnormalities, such as hypopigmentation. In recent years, dynamic analyses by more precise microscopic observations have revealed specific functions of a variety of molecules involved in melanogenesis. This review article focuses on the latest findings with regard to the steps (or mechanisms) involved in melanosome formation and transport of mature melanosomes within epidermal melanocytes. Finally, we will touch on current topics in melanosome research, particularly on the "melanosome transfer" and "post-transfer" steps, and discuss future directions in pigment research.

## Introduction

Melanin is the major pigment in mammalian skin and hair and is synthesized in specialized cells called melanocytes, which are present in the epidermis and in the matrix and outer root sheath of hair follicles. Melanin is present in the form of polymers formed from various indole compounds synthesized from the amino acid tyrosine. Two types of melanin are found in mammals: insoluble black eumelanin and soluble yellow-reddish pheomelanin. Mammalian melanin is a complex of these two types of melanin, and their ratios are responsible for the differences in skin and hair color. Tyrosine is oxidized by the copper-containing enzyme tyrosinase and metabolized to dihydroxyphenylalanine (DOPA) and then to dopaquinone. Eumelanin and pheomelanin are formed by different metabolic pathways downstream of dopaquinone ^1^. Since many of the intermediate molecules leading to melanin formation possess high redox toxicity, melanin synthesis is sequestered and executed in a specialized compartment, lysosome-related organelles called melanosomes ^2, 3^. Strictly speaking, melanogenesis refers to the process of melanin synthesis, but, more broadly, it includes the processes of melanosome formation, melanosome transport, melanosome transfer to keratinocytes, and melanin metabolism because these processes are also necessary for skin and hair pigmentation.

Melanosomes are classified into four stages according to their morphology and degree of pigment deposition ^3^. Stage I melanosomes are typically early/sorting endosomes with clathrin coats and contain few intraluminal vesicles (ILVs). Physiological amyloid fibers are formed from ILVs by polymerization of processed premelanosome protein (PMEL), and they provide ellipsoidal shape to melanosomes and also serve as a scaffold for melanin pigmentation (stage II). Melanogenic enzymes, including tyrosinase, tyrosinase-related protein 1 (Tyrp1), and dopachrome tautomerase (Dct)/Tyrp2, are then transported to stage II melanosomes, and the melanosomes darken as melanin deposits on PMEL fibers (stages III and IV) ^2, 3^. This review article summarizes recent research topics related to melanosome formation and movements, with a special focus on the past 4 years.

## Melanosome biogenesis

**Figure 1.**
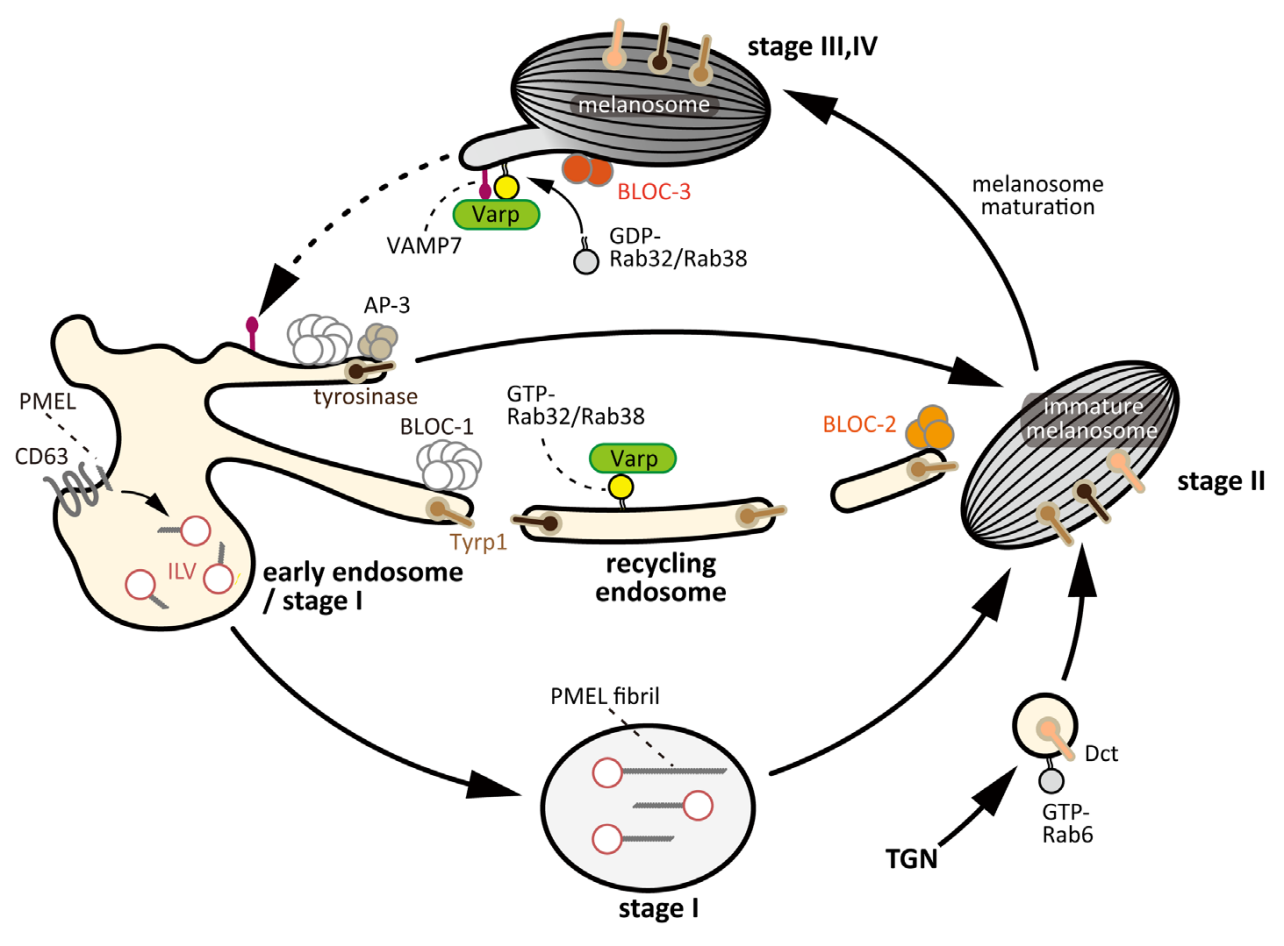
Schematic diagram of endosomal and melanosomal transport systems that have been proposed to regulate melanosome biogenesis. PMEL fibrils emanate from ILVs in the vacuolar portion of early endosomes, a process that requires the functions of CD63 and apolipoprotein E. The PMEL fibrils then form sheets onto which synthesized melanin is deposited. Melanosomal cargoes, including tyrosinase and Tyrp1, are secreted from the microdomains of early endosomes. Rab4A is postulated to regulate the segregation of the melanogenic enzymes from the vacuolar portion of the early endosomes ^15^ (not depicted in this figure). The BLOC-1/2-dependent pathway regulates the transport of Tyrp1 and a small cohort of tyrosinase, and the AP-3-dependent pathway regulates the transport of a large cohort of tyrosinase. BLOC-1 is an important regulator in the formation of transport carriers in a microtubule- and actin-dependent manner. BLOC-2 is involved in the fusion of transport carriers with maturing melanosomes, and BLOC-3 is required for the activation of Rab32/Rab38 on mature melanosomes. The Rab32/Rab38–Varp complex is required for the recovery of melanosomal cargoes, such as VAMP7, from mature melanosomes (retrograde trafficking), and is also required for the transport of melanogenic enzymes from early endosomes to maturing melanosomes (anterograde trafficking). The dotted arrow indicates an as-yet-unidentified transport pathway. AP, adaptor protein; BLOC, biogenesis of lysosome-related organelles complex; ILV, intraluminal vesicle; PMEL, premelanosome protein; TGN, *trans*-Golgi network; Tyrp1, tyrosinase-related protein 1; VAMP, vesicle-associated membrane protein; Varp, VPS9-ankyrin-repeat protein.

Transport of PMEL (also known as Pmel17 or gp100), a membrane protein, to immature melanosomes is an early step in melanosome biogenesis, but the mechanism by which it is achieved is not completely understood. PMEL is taken into the lumen of stage I immature melanosomes and proteolytically cleaved, a process that is required to form the fibrils that emanate from ILVs ( Figure 1). This process requires the tetraspanin CD63, apolipoprotein E, and BACE2 (β-secretase 2) ^4, 5^. After PMEL fibril formation, the melanogenic enzymes are transported from the *trans*-Golgi network (TGN) or early endosomes to stage II immature melanosomes, and the melanin synthesized accumulates on the PMEL fibrils. The melanogenic enzymes are transported to the immature melanosomes via two endosomal pathways: a biogenesis of lysosome-associated organelles complex (BLOC)-1-dependent pathway and a BLOC-1-independent/adaptor protein-3 (AP-3)-dependent pathway, although BLOC-1 is also physically and functionally associated with the AP-3-dependent pathway ^2, 6^. At steady state, BLOC-1 and AP-3 are thought to localize at distinct microdomains of early endosomes and to transport only partially overlapping melanosomal cargoes. BLOC-1 is a protein complex composed of eight subunits, including BLOC1 subunit 1 (BLOS1), BLOS2, BLOS3, Cappuccino/BLOC1S4, Muted/BLOC1S5, Pallidin/BLOC1S6, Snapin/BLOC1S7, and Dysbindin/BLOC1S8, that is initially localized on early endosomes and is essential for the formation of the transport carriers for melanosomal cargoes ^3^. BLOC-1 has been shown to associate with the kinesin-3 motor and promote microtubule-dependent tubule formation, and then to cooperate with annexin A2 to form recycling endosomes by rearrangement of the actin cytoskeleton. Three of the BLOC1 subunits, BLOS1, BLOS2, and Snapin, are also present in another complex, BLOC-one-related complex (BORC), which regulates lysosomal centrifugal transport ^7^, but the functions of BORC in melanogenesis remain to be determined ^8^. UV radiation resistance-associated gene (UVRAG) is an adapter molecule that promotes autophagy and regulates energy homeostasis ^9^, and it is also known to be involved in vitiligo ^10^. UVRAG has recently been shown to bind to and stabilize several subunits of BLOC-1 (e.g. BLOS1 and Snapin), and melanosomal cargoes, such as tyrosinase and Tyrp1, are mis-trafficked and accumulate at early endosomes in UVRAG-deficient melanocytes. PMEL is also mis-trafficked in UVRAG-deficient melanocytes, but the mechanism of UVRAG-mediated PMEL trafficking is unknown ^11^. However, since the UVRAG-regulated BLOC1 subunits are also shared with BORC, further investigation is necessary to determine whether the UVRAG’s role in melanosome cargo transport requires BLOC-1 or BORC.

Melanosomes are organelles that possess dynamic properties, and their lipid component is postulated to be involved in their membrane dynamics. Actually, melanosomes contain the phospholipid phosphatidylinositol 3,5-bisphosphate—PI(3,5)P _2_—which is specifically present in late endosomes/multivesicular bodies and lysosomes and regulates membrane trafficking between endo-lysosomes ^12^. PI(3,5)P _2_ is synthesized from PI(3)P by the phosphoinositide 5-kinase complex, which is composed of phosphoinositide kinase, FYVE-type zinc finger containing (PIKfyve), FIG4, and VAC14, and abnormal metabolism of PI(3,5)P _2_ has been shown to be involved in hypopigmentation. A recent study reported that PIKfyve regulates the fusion of stage I melanosomes with lysosomes, a process that is involved in melanosome quality control ^13^, and another group of researchers showed that PIKfyve regulates the trafficking of melanosomal cargoes to melanosomes as well as PMEL processing ^14^. These findings suggest a role for PIKfyve in the formation of PMEL fibers and its organization in stage II melanosomes.

BLOC-2 is a protein complex composed of three subunits (HPS3, HPS5, and HPS6; the gene products responsible for Hermansky–Pudlak syndrome 3, 5, and 6, respectively). BLOC-2 localizes on early endosomes and on transport carriers like BLOC-1 and is postulated to function downstream of BLOC-1. Although the precise function of BLOC-2 remains unknown, BLOC-2 is thought to tether the transport carriers to the melanosome membrane ^16^. In addition, one of the endosomal Rabs, Rab22A, has been reported to promote the formation of the transport carriers from early endosomes by forming a complex with BLOC-1, BLOC-2, and the kinesin-3 motor ^17^.

**Figure 2.**
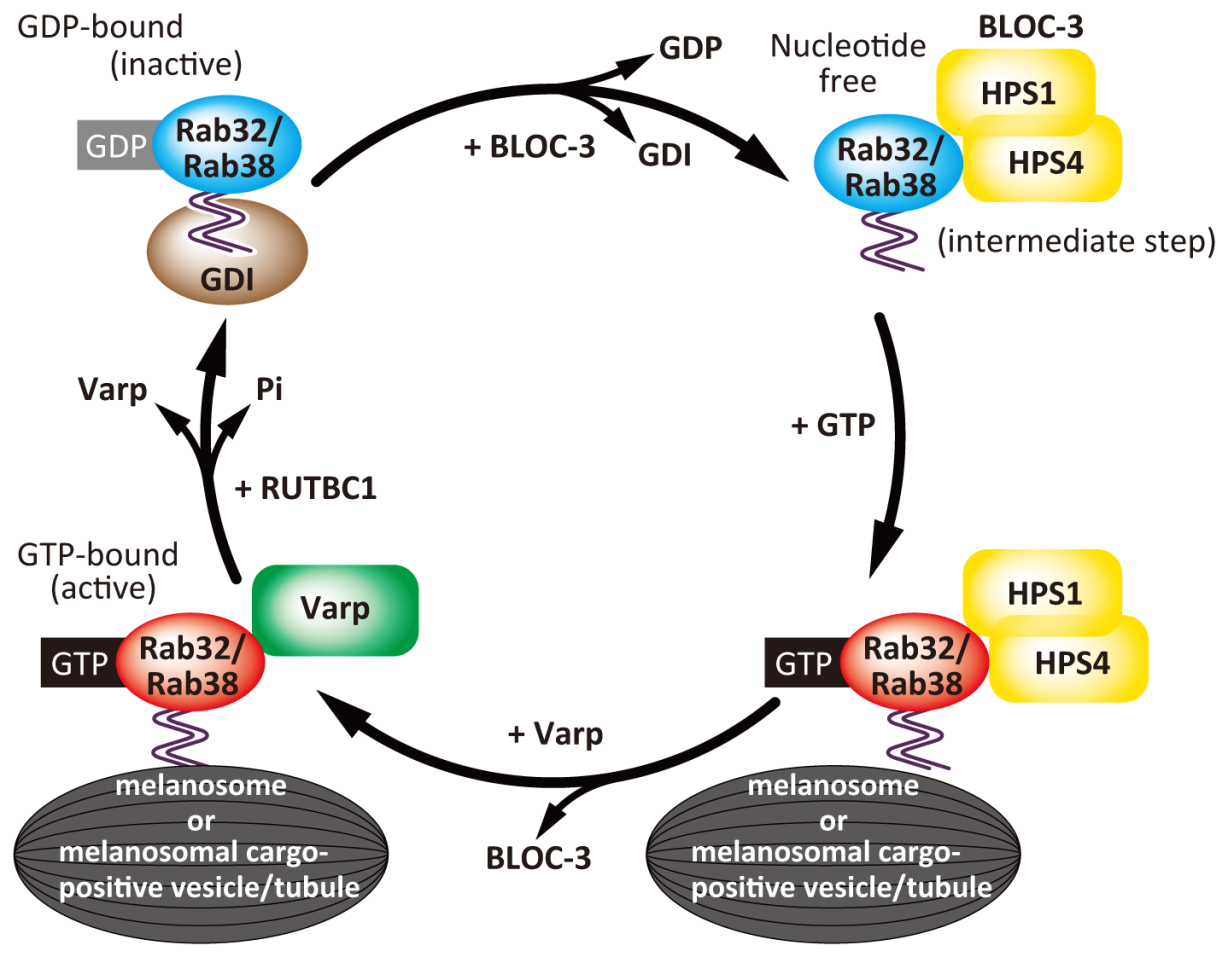
Diagram of Rab activation and inactivation during membrane traffic. Rabs are a subgroup of Ras-like small GTPases and are switch molecules that cycle between a GDP-binding inactive form and a GTP-binding active form. In this figure, Rab activation and inactivation mechanisms are shown by using Rab32 and Rab38 as examples. GDP-binding inactive Rab32 and Rab38 are present in the cytosol in complex with a GDI. BLOC-3 is a Rab32/Rab38-GEF that removes GDP from the GDP-Rab32/Rab38 and promotes their GTP binding, which converts Rab32/Rab38 to their active form. At the same time, Rab32/Rab38 are localized to the intracellular membrane surface by means of a geranylgeranyl group attached to the C-terminal cysteine residues of Rab32/Rab38. Active Rab32/Rab38 interact with one of their effector molecules, Varp, and regulate membrane trafficking, such as melanosomal cargo transport. The Rab32/Rab38-GAP RUTBC1 then activates the intrinsic GTPase activity of Rab32/Rab38, thereby hydrolyzing their GTP, and returning them to inactive GDP-Rab32/Rab38. BLOC, biogenesis of lysosome-related organelles complex; GAP, GTPase-activating protein; GDI, GDP dissociation inhibitor; GEF, guanine nucleotide exchange factor; HPS, Hermansky–Pudlak syndrome; RUTBC1, RUN and TBC1 domain-containing protein 1; Varp, VPS9-ankyrin-repeat protein.

Other subsets of Rabs with similar primary structures, Rab32 and Rab38 ^18^, are localized to the melanosome membrane and redundantly regulate melanosome biogenesis ^19^. Rab32 and Rab38 are switch molecules that cycle between an active GTP-binding and an inactive GDP-binding form, the same as other Rabs ( Figure 2). BLOC-3 is a heterodimeric protein complex composed of HPS1 and HPS4 and functions as a Rab32/Rab38-guanine nucleotide exchange factor (GEF) ^20^. In addition, HPS4 has been reported to be a potential effector molecule of Rab9 ^21^. The Rab9–BLOC-3 axis was postulated to be involved in melanogenesis ^22^, but it has been shown that the Rab32/Rab38-GEF activity of BLOC-3 is essential for melanogenesis and the Rab9 binding activity is not ^23^. The Rab32/Rab38 effector vacuolar protein sorting 9 (VPS9)-ankyrin repeat protein (Varp) is never recruited to melanosomes in BLOC-3-deficient melanocytes, and the trafficking of melanogenic enzymes, including tyrosinase, Tyrp1, and Dct, is impaired as a result ^2, 23– 26^. The Rab32/Rab38–Varp complex has been thought to regulate anterograde transport of tyrosinase and Tyrp1 from early endosomes to melanosomes ^27^; however, it has been shown to be utilized to recover another melanosomal cargo vesicle-associated membrane protein 7 (VAMP7) from mature melanosomes by cooperating with myosin VI ^25^ ( Figure 1). The recovered VAMP7 is postulated to return to early endosomes for the next round of melanogenic enzyme transport. Rab32/Rab38 cycling is essential for the proper trafficking of melanogenic enzymes because the Rab32/Rab38-GTPase-activating protein (GAP) RUN and TBC1 domain-containing protein 1 (RUTBC1 [also known as small G-protein signaling modulator 2 (SGSM2)]) promotes melanogenic enzyme trafficking to melanosomes ^26^. Varp protein expression is considered to be essential for the correct transport of the melanogenic enzymes, and Rab40C and receptor for activated C kinase 1 (RACK1) have been shown to precisely regulate the amount of Varp via direct binding to the ankyrin repeat 2 (ANKR2) domain of Varp in a ubiquitination-dependent manner ^28– 30^. The involvement of Rab32/Rab38 in Dct trafficking appears to vary with the cell line ^26, 31^. Rab6 and its effector molecule, ELKS, have recently been suggested to mediate a pathway that may directly transport Dct from the TGN to melanosomes ^32^. Future studies will elucidate how Rab32/Rab38 are required for Dct transport and the functional relationship between Rab32/Rab38- and Rab6-dependent pathways.

In addition to melanosomal structural proteins and melanogenic enzymes, certain transporters (or ion channels) are also required for melanogenesis to optimize luminal pH and concentration of other ions (e.g. Cu ^2+^ and Zn ^2+^) in melanosomes. Recently, functions of several transporters localized to the melanosome membrane have been revealed. Oculocutaneous albinism 2 (OCA2) and solute carrier 45 member 2 (SLC45A2, also known as MATP, membrane-associated transporter protein, or OCA4) are known to be the causative gene products of oculocutaneous albinism ^33, 34^. From fine analysis using a patch-clamp technique and a pH sensor protein on the melanosome membrane, OCA2 has been proposed to promote anion (e.g. Cl ^–^) efflux from melanosomes to elevate their luminal pH (i.e. neutralization) ^33^. SLC45A2 has also been shown to be involved in the neutralization of melanosomal pH by promoting H ^+^-efflux from melanosomes ^34^. Because tyrosinase is inactive below pH 6, OCA2 and SLC45A2 are thought to be essential transporters for promoting pigmentation. Two-pore channel 2 (TPC2) has also been associated with human pigmentation diseases ^35^. TPC2 is postulated to pass cations such as Na ^+^ and Ca ^2+^ across the melanosome membrane, and it has been shown that suppressing TPC2’s function promotes pigmentation by raising the luminal pH in melanosomes ^36, 37^. Although which cation(s) transported by TPC2 is responsible for acidification of melanosomes remains to be determined, Na ^+^ or Ca ^2+^ efflux-mediated increase of melanosomal membrane potential has been proposed to modulate the function of vacuolar-type H ^+^-ATPase (V-ATPase) to acidify melanosomes ^36, 37^. Thus, proper pigmentation would be achieved by the cooperative action of the neutralizing transporters (e.g. OCA2 and SLC45A2) and the acidifying transporters (e.g. TPC2) on the melanosome membrane.

## Melanosome movements in melanocytes

**Figure 3.**
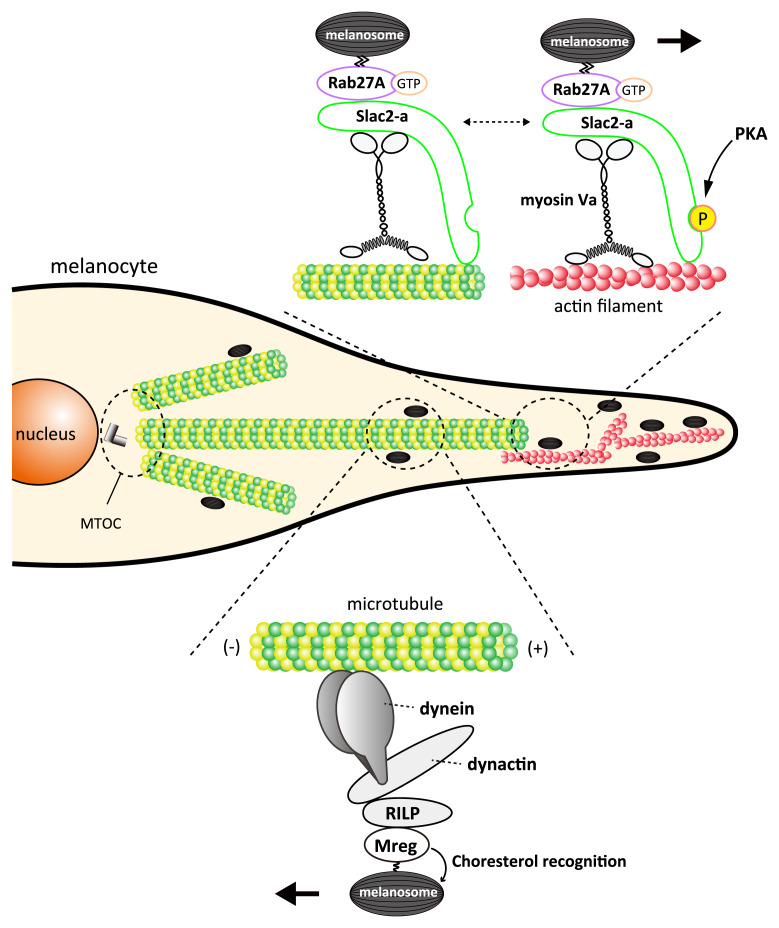
Mechanisms of mature melanosome transport along the cytoskeleton in melanocytes. Coordinated bidirectional movements along microtubules and unidirectional movement along actin filaments regulate the distribution of mature melanosomes within melanocytes, and the balance between microtubule-dependent retrograde transport and actin-dependent anterograde transport, in particular, is essential to the intracellular distribution of melanosomes. Mreg and Rab36 ^40^ (not depicted in this figure) together with RILP, dynactin, and dynein play important roles in microtubule-dependent retrograde transport ^41^, and the Rab27A–Slac2-a/MLPH–myosin Va complex plays an important role in actin-dependent anterograde transport. PKA mediates the phosphorylation of Slac2-a/MLPH and facilitates the Slac2-a/MLPH interaction with actin filaments, thereby promoting the actin-dependent melanosome transport ^42^. In addition, phosphorylated Slac2-a/MLPH by PKA would also suppress the function of the dynein motor ^43^. MLPH, melanophilin; Mreg, melanoregulin; PKA, protein kinase A; RILP, Rab-interacting lysosomal protein.

The intracellular localization of mature melanosomes is regulated by coordinated transport activity in opposite directions on microtubules (i.e. long-range anterograde and retrograde microtubule-dependent transport) as well as short-range anterograde transport on actin filaments ^2, 38^. Here we describe some recent findings with regard to the regulation of melanosome transport ( Figure 3). Melanosome capturing by actin filaments and subsequent actin-based melanosome transport are regulated by a tripartite protein complex, Rab27A–Slac2-a (also known as melanophilin [MLPH])–myosin Va, and abnormalities in the function of this complex cause Griscelli syndrome, a rare autosomal recessive hypopigmentation disease ^2, 38^. MAP kinase activating death domain (MADD, also known as Rab3GEP or differentially expressed in normal and neoplastic cells [DENN]) had already been reported to be a Rab27A-GEF in melanocytes ^39^. Re-analysis by the same group, however, showed that MADD/Rab3GEP/DENN functions as a Rab27A-GEF but is insufficient to activate Rab27A. Other Rab-GEFs, such as DENND4B and GRAB, presumably compensate for the function of MADD/Rab3GEP/DENN ^44^.

Melanoregulin (Mreg) is a small protein with no conserved protein motifs and has been shown to be involved in retrograde melanosome transport along microtubules and in melanosome transfer to keratinocytes ^41, 45^. Its three-dimensional structure has recently been determined, and a tyrosine-based cholesterol recognition motif (CRAC motif) in Mreg has been found to be important to the retrograde transport of melanosomes along microtubules. Importantly, an Mreg mutant that lacks the CRAC motif does not promote the retrograde transport of melanosomes ^46^. However, the precise molecular mechanism by which switching from microtubule-dependent to actin-dependent melanosome transport is regulated remains poorly understood. Phosphorylation of Slac2-a/MLPH may be one potential regulatory mechanism because, as a result of phosphorylation by protein kinase A (PKA), Slac2-a/MLPH has been shown to facilitate its interaction with actin filaments and PKA is important in determining the subcellular localization of melanosomes ^42, 47^. Moreover, zebrafish Slac2-a/MLPH has been suggested to suppress the function of dynein motor by cAMP- and PKA-dependent phosphorylation ^43^. Thus, it is also interesting to investigate the functional regulation of molecules involved in retrograde melanosome transport along microtubules (e.g. Mreg, Rab-interacting lysosomal protein [RILP], dynactin, and dynein, depicted in Figure 3) by phosphorylation in mammalian melanocytes in the future.

## Perspectives

We have summarized recent findings with regard to melanosome formation and movements in mammalian melanocytes. Fairly precise mechanisms for these events have gradually been demonstrated by recent elegant work conducted by various laboratories. However, achieving a comprehensive understanding of pigmentation in the broad sense will require elucidation of the mechanisms involved in the melanosome transfer process (i.e. the process of transferring melanosomes from melanocytes to keratinocytes or hair matrix cells) and melanin metabolism in keratinocytes, neither of which is fully understood. To date, at least four different models have been proposed for the melanosome transfer process: (i) melanosome-enriched dendrites of melanocytes, (ii) melanosome aggregates shed from dendrites, or (iii) exocytosed melanocores that are contained in melanosomes (i.e. melanosomes without their limiting membrane) are incorporated into keratinocytes by phagocytosis and/or endocytosis, and (iv) the two membranes of a melanocyte and a keratinocyte fuse, and melanosomes are transferred through the tubular structure formed between them. These models are summarized in recent reviews ^48– 50^. Live-cell bright-field observations and/or electron microscopic observations of melanocore and melanosome dynamics have been conducted to test these models, but bright-field imaging has the drawback of very low resolution of the spatial distribution of melanosomes, and electron microscopy can be performed on only fixed cells and thus cannot be used to make dynamic observations. Labeling melanin with a fluorescent probe should be a useful tool for making more accurate analyses of melanocore and melanosome dynamics in melanocytes and keratinocytes. Fortunately, we have been developing a fluorescent probe, melanocore-interacting Kif1c-tail (M-INK), that specifically recognizes melanocores and makes it possible to visualize the spatial distribution of incorporated melanosomes and melanocores in keratinocytes ^51^. Using this probe has recently made it possible to identify Rab7B (also known as Rab42) as a key regulator that promotes melanosome protein degradation in keratinocytes ^52^. Thus, the M-INK probe should be a useful tool for analyzing the metabolic pathway(s) of melanin in keratinocytes in the future.

## Abbreviations

AP, adaptor protein; BLOC, biogenesis of lysosome-related organelles complex; BLOS, BLOC1 subunit; BORC, BLOC-one-related complex; DENN, differentially expressed in normal and neoplastic cells; Dct, dopachrome tautomerase; GEF, guanine nucleotide exchange factor; HPS, Hermansky–Pudlak syndrome; ILV, intraluminal vesicle; MADD, MAP kinase activating death domain; M-INK, melanocore-interacting Kif1c-tail; MLPH, melanophilin; Mreg, melanoregulin; OCA2, oculocutaneous albinism 2; PKA, protein kinase A; PIKfyve, phosphoinositide kinase, FYVE-type zinc finger containing; PI(3,5)P _2_, phosphatidylinositol 3,5-bisphosphate; PMEL, premelanosome protein; SLC45A2, solute carrier 45 member 2; TPC2, two-pore channel 2; TGN, *trans*-Golgi network; Tyrp1, tyrosinase-related protein 1; UVRAG, UV radiation resistance-associated gene; VAMP, vesicle-associated membrane protein; Varp, vacuolar sorting protein 9-ankyrin-repeat protein.
